# The heart failure burden of type 2 diabetes mellitus—a review of pathophysiology and interventions

**DOI:** 10.1007/s10741-018-9685-0

**Published:** 2018-03-08

**Authors:** Anne Pernille Ofstad, Dan Atar, Lars Gullestad, Gisle Langslet, Odd Erik Johansen

**Affiliations:** 1 Bærum Hospital, Vestre Viken HF, Rud, Norway; 2 Medical Department, Boehringer Ingelheim, Asker, Norway; 3 Department of Cardiology B, Oslo University Hospital, Ullevål, Oslo, Norway; 4 Faculty of Medicine, University of Oslo, Oslo, Norway; 5 Department of Cardiology, Oslo University Hospital, Rikshospitalet, Oslo, Norway; 6 Rikshospitalet, Lipid Clinic, Oslo University Hospital, Oslo, Norway

**Keywords:** Heart failure, Diabetes mellitus, Review, Glucose lowering

## Abstract

Diabetes and heart failure (HF) are both global epidemics with tremendous costs on society with increased rates of HF hospitalizations and worsened prognosis when co-existing, making it a significant “deadly duo.” The evidence for pharmacological treatment of HF in patients with type 2 diabetes mellitus (T2DM) stems typically from either subgroup analyses of patients that were recruited to randomized controlled trials of HF interventions, usually in patients with reduced ejection fraction (EF), or from subgroup analyses of HF patients recruited to cardiovascular (CV) outcome trials (CVOT) of glucose lowering agents involving patients with T2DM. Studies in patients with HF with preserved EF are sparse. This review summarizes the literature on pathophysiology and interventions aiming to reduce the HF burden in T2DM and includes HF trials of ACEi, digoxin, β-blocker, ARB, I_f_-blocker, MRA, and ARNI involving 38,600 patients, with or without prevalent diabetes, and CV outcome trials in T2DM involving 74,351 patients, with or without prevalent HF. In all HF trials, HF outcomes by prevalent diabetes were reported with an incremental risk of HF and death confessed by prevalent diabetes and a treatment effect similar to those without diabetes. All T2DM CVOTs reported on HF outcomes with heterogeneity between trials with two reporting benefits (empagliflozin and canagliflozin) and two reporting increased risk (saxagliptin, pioglitazone). In vulnerable T2DM patients with concomitant HF, guideline-recommended HF drugs are effective. When choosing glucose-lowering therapy, outcomes from available CVOTs should be considered.

## Introduction

Heart failure (HF) can be defined as a complex clinical syndrome that results from any structural or functional impairment of ventricular filling or ejection of blood [1]. It is a global pandemic affecting more than 26 million people worldwide [2] and in developed countries approximately 1–2% of the adult population. Its prevalence increases with age and comorbidities such as hypertension, obesity, and type 2 diabetes mellitus (T2DM) [3], and this has implications given the diabetes and obesity (i.e., “diabesity”) epidemic we currently are facing [4]. Although improved evidence-based treatment has led to improved survival [5, 6], the 5-year mortality rate in advanced HF is approximately 50% [6], and in some countries, the number of deaths from HF has surpassed the number of deaths from myocardial infarction [7]. According to HF registries and clinical trials, patient characteristics, demographics, and treatment traditions in HF vary across geographical regions [2, 8], and this may cause challenges in the interpretation of the study results.

Alongside the projected increase in prevalence, a tremendous impact on societal costs is expected, as illustrated by US projections showing that by 2030, the total cost of HF will increase almost 127% to $ 69.7 billion from 2012 (when it was $30.7 billions) [9]. Effective preventive measures are therefore needed that can address the expected increased burden of HF. An area where attention in particular is needed is in patients with concomitant T2DM and HF where recent data suggest an incremental risk of cardiovascular (CV) death and hospitalization for HF, as compared to patients with HF without T2DM [10]. This review discusses the epidemiology and pathophysiology of HF in T2DM, as well as the existing evidence for treatment and prevention of HF in T2DM, including effects of specific glucose-lowering drugs.

## Methods

This review is based on a literature search in PubMed or MEDLINE, or at the scientific conference websites of major international cardiology (e.g., European Society of Cardiology (ESC), ESC HF association, American College of Cardiology (ACC) or American Heart Association (AHA)) or diabetes (i.e., European Association for the Study of Diabetes (EASD) or American Diabetes Association (ADA)) societies until June 12th 2017. We review the current pathophysiological understanding as well as contemporary placebo-/comparator-controlled HF trials and well powered CV outcome studies of glucose-lowering drugs in T2DM. We include studies of major drug classes in HF guidelines (angiotensin converting enzyme inhibitors (ACEis), β-blockers, angiotensin receptor blockers (ARBs), I_f_-blocker, mineralocorticoid receptor antagonists (MRA), angiotensin receptor-neprilysin inhibitor (ARNI) and digoxin) that have reported on outcomes of HF interventions in patients with or without prevalent T2DM, as well as CV outcome trials that have reported on HF outcomes in T2DM patients with or without prevalent HF. We excluded any studies where the results in patients with co-existing HF and T2DM were not reported (e.g., The Randomized Aldactone Evaluation Study (RALES) and The Cooperative North Scandinavian Enalapril Survival (CONSENSUS) study), or trials that compared low versus high dose of the same intervention (e.g., the Assessment of Treatment with Lisinopril And Survival (ATLAS) study of high versus low dose of lisinopril), or T2DM studies that mainly tested intensive versus conventional glucose-lowering strategies (e.g., Veterans Affairs Diabetes Trial (VADT), The Action to Control Cardiovascular Risk in Diabetes (ACCORD) trial, The Action in Diabetes and Vascular Disease: Preterax and Diamicron Modified Release Controlled Evaluation (ADVANCE) trial). We report the characteristics of the trials as well as the major HF outcomes and the annualized incidence rates (reported or derived based on number/proportions of patients with event and median or mean follow-up time) or the absolute proportion of patients in the trials with HF event.

## Results and discussion

### Epidemiology and prognosis of HF in T2DM

**Fig. 1:**
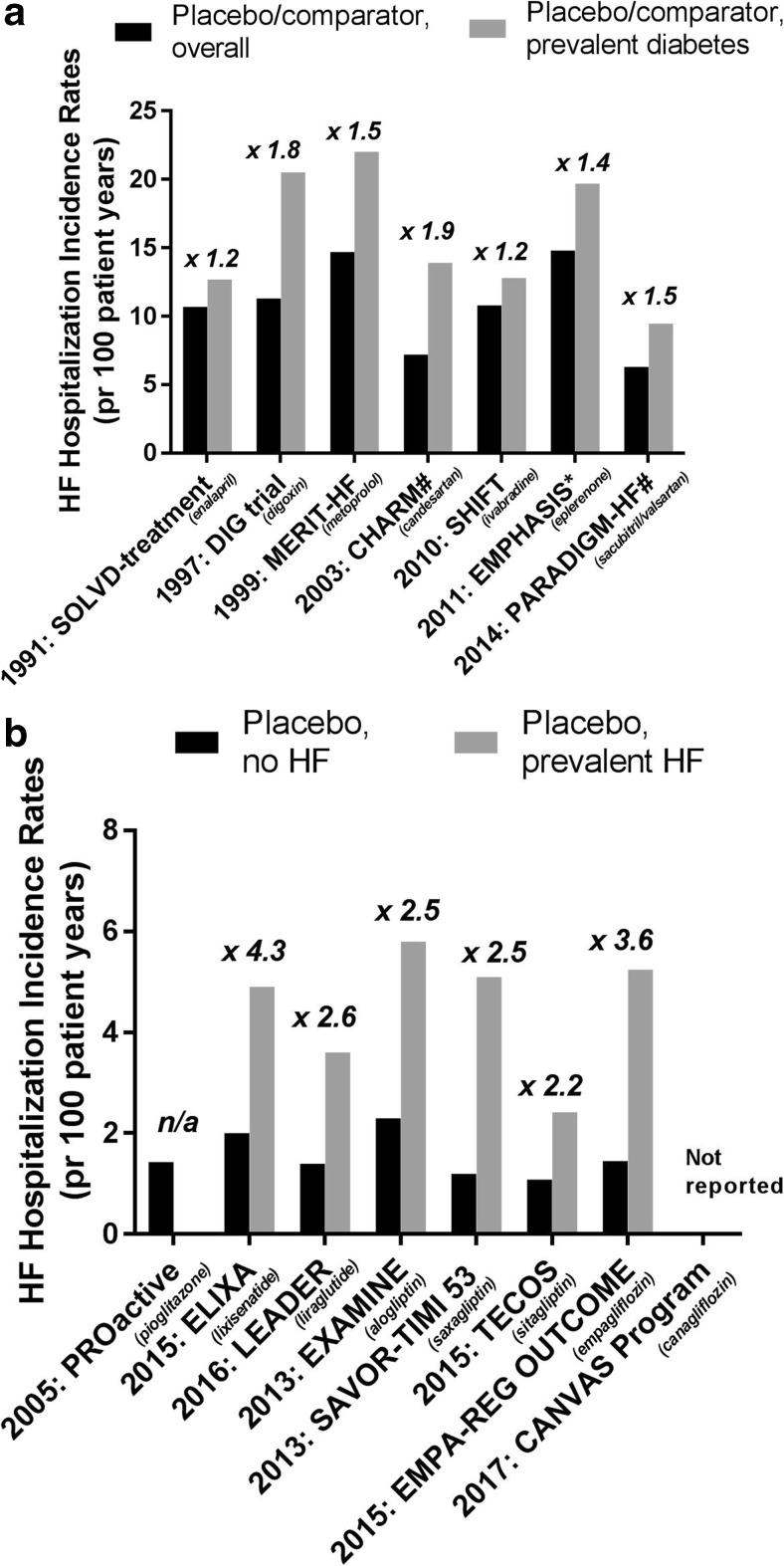
**a** Incidence of HF hospitalization in the overall and DM subgroup in placebo/comparator-arms of HF trials of different interventions (ACEi [13, 17], digoxin [18, 19], β-blocker [20, 21], ARB [10, 22], I_f_-blocker [23, 24], MRA [25, 26], and ARNI [27, 28]) and the relative incidence rate ratio for HF hospitalization for prevalent DM vs no DM. #: incidence rates in the overall groups (comparator + active), *: incidence rates include CV death. Abbreviations: HR: hazard ratio, HF: heart failure, SOLVD: Studies of Left Ventricular Dysfunction, DIG-trial: The Digitalis Investigation Group (DIG) trial, MERIT-HF: Metoprolol CR/XL Randomized Intervention Trial in Congestive Heart Failure, CHARM: Candesartan Assessment of Reduction in Mortality and morbidity, SHIFT: The Systolic Heart Failure Treatment With the *I*_f_ Inhibitor Ivabradine Trial, EMPHASIS: Eplerenone in Mild Patients Hospitalization and Survival Study in Heart Failure, PARADIGM-HF: Prospective Comparison of ARNI with ACEI to Determine Impact on Global Mortality and Morbidity in Heart Failure. **b** Incidence of HF hospitalization by prevalent HF in placebo arms of CV outcomes trials of glucose-lowering drugs (pioglitazone [29, 30], lixisenatide [31, 32], liraglutide [33, 34], alogliptin [35, 36], saxagliptin [37, 38], sitagliptin [39, 40], and empagliflozin [41, 42]) and the relative incidence rate ratio for HF hospitalization for prevalent HF vs no HF. Abbreviations: HF: heart failure, CV: cardiovascular, n/a: not applicable, PROactive: PROspective pioglitAzone Clinical Trial In macroVascular Events, ELIXA: the Evaluation of LIXisenatide in Acute Coronary Syndrome trial, LEADER: the Liraglutide Effect and Action in Diabetes: Evaluation of cardiovascular outcome Results—a long term evaluation trial, EXAMINE: The Examination of Cardiovascular Outcomes with Alogliptin versus Standard of Care, SAVOR-TIMI 53: The Saxagliptin Assessment of Vascular Outcomes Recorded in Patients with Diabetes Mellitus 53 trial, TECOS: The Trial Evaluating Cardiovascular Outcomes With Sitagliptin, CANVAS: CANagliflozin cardioVascular Assessment Study

The Framingham study reported already in 1974 that men and women with diabetes mellitus (DM) had a 2-fold and 5-fold increased risk, respectively, of incident HF during 18 years of follow-up, as compared to non-diabetic men and women [11]. This has later been confirmed in various studies, e.g., the Reykjavik study which reported a 12% prevalence of HF among the population with DM, and only 3% among those without DM [12]. HF and concomitant T2DM is a strong predictor for adverse outcomes [13] and is associated with an approximately 2-fold higher risk for CV or all-cause mortality [14–16]. Thus, an incremental HF burden with co-existing DM is apparent. As illustrated in Fig. 1a, this is observed both in major HF trials of drugs tested to prevent or treat HF (ACEi, digoxin, β-blocker, ARB, If-blocker, MRA, ARNI), and, as illustrated in Fig. 1b, in major CV outcome trials testing glucose-lowering drugs (pioglitazone, GLP-1 receptor analogues, DPP-4 inhibitors, SGLT-2- inhibitors). In fact, the incremental risk for being hospitalized for HF by concomitant DM in dedicated HF trials is 1.2–1.9-fold, whereas the excess rate for HF hospitalizations by concomitant HF in dedicated diabetes trials is 2.2–4.3-fold. Interestingly, the same magnitude of excess risk seems to hold true in patients with T2DM who have HF with reduced EF (HFrEF), as well as in those with HF with *preserved* EF (HFpEF), as seen in the “Candesartan Assessment of Reduction in Mortality and morbidity (CHARM) programme” [10].

### Pathophysiology of HF in T2DM

The pathophysiology for HF development in T2DM is complex. Beside the general HF risk factors like advancing age, ethnicity, genetic predisposition, hypertension, and smoking, T2DM increases the risk of ischemic HF through increased risk of coronary artery disease (CAD), as well as impacting directly the myocardium leading to structural and functional changes (“diabetic cardiomyopathy”) [43, 44]. The hallmark of T2DM, namely hyperglycemia, is a major contributor, and observational data suggest an 8–16% increased risk of HF for each 1%-point increase in HbA1c [45, 46]. Furthermore, T2DM is associated with obesity and visceral adiposity (e.g., epicardial adipose tissue) which is associated with impaired myocardial function [47] and an increased risk of HF [48]. Additionally, T2DM is associated with an accelerated decline in renal function and increased risk for chronic kidney disease, which adversely influences risk for HF outcome [49].

The atherosclerotic processes are accelerated in T2DM with more lipid-rich and unstable atherosclerotic plaques as compared to non-diabetic atherosclerosis [50], leading to a 2–4 times increased risk for CV morbidity and mortality [51–53]. In the setting of DM, the outcome after MIs is poorer with increased risk of re-infarction [54], re-hospitalization for HF [55], and complicating HF [56], potentially caused by the poorer formation of collateral vessels in response to ischemia [57], with endothelial dysfunction suggested as a central underlying mechanism.

Endothelial dysfunction and microangiopathic processes might also be important for the development of diabetic cardiomyopathy [58]. However, a unifying explanation remains to be fully elucidated [44, 59, 60] considering multiple interrelated factors including hyperglycemia and elevated levels of free fatty acids as observed in T2DM. These induce a shift in substrate metabolism leading to increased formation of reactive oxygen species that promote cardiac remodeling and impair myocardial contractility. In addition comes the formation of advanced glycation end products, upregulation of the renin-angiotensin-aldosterone system (RAAS), and probably intramyocardial inflammation contributing to the increased myocardial stiffness, impaired energy availability, and reduced contractility seen in early diabetic cardiomyopathy. The presence of subclinical diabetic cardiomyopathy makes the heart more vulnerable and incompetent to respond to stress and ischemia and is believed, at least in part, to contribute to the worsened outcome in T2DM following coronary events [61, 62] and the increased risk of developing overt HF [63].

### Characteristics of HF in T2DM

Structural cardiac changes seen in T2DM include increased interstitial fibrosis, increased left ventricular (LV) wall thickness, and often increased LV mass [44, 64], alterations which contribute to, but are not a prerequisite to, the development of functional myocardial impairments. As a consequence, diastolic dysfunction is the classical and most frequent early cardiac functional abnormality in T2DM patients [44], and asymptomatic diastolic dysfunction has been detected in up to 75% of normotensive DM patients without evident CAD [65]. With increasing availability of more sophisticated imaging techniques, the presence of subtle systolic alterations, i.e., decreased deformation indices such as strain and strain rate, have become apparent and often seem to develop as early as the diastolic impairment [66]. The myocardial dysfunction in T2DM usually is progressive with an early asymptomatic phase where the heart hypertrophies, leading to diastolic dysfunction in the setting of preserved LV ejection fraction (LVEF) [59]. This is followed by a late stage, which is characterized by alteration in microvasculature compliance, an increase in left ventricular size, and a decrease in cardiac performance leading to symptomatic HF. Predictors for progression to late stage, which may take several years, include comorbidities often seen in T2DM such as CAD, hypertension, obesity, and microvascular changes [67].

### HFrEF vs HFpEF in T2DM

HF is categorized according to LVEF ≤ 40% (HFrEF) or > 50% (HFpEF). This terminology is useful due to the prognostic importance of EF in HF, as illustrated by the higher relative risk of CV death or HF hospitalization seen in the CHARM study program by presence of T2DM and HFrEF versus T2DM and HFpEF [10]. Further, major HF trials selected patients based on EF, and evidence suggests that HFpEF and HFrEF might be distinct entities with different pathophysiological mechanisms and different responses to treatment [68–70]. As we discuss below, the mean prevalence of T2DM across the large HF trials is 27% (Tables 1 and 2), independent of EF range studied.

**Table 1:** Key features, DM prevalence, and treatment effect on HF outcomes in the overall study population and by prevalent DM in the large clinical HF trials involving ACEis, β-blockers, and a MRA

Study	SOLVD treatment	MERIT-HF	CIBIS-II	COPERNICUS	EMPHASIS
Year completed	1991	1999	1999	2001	2011
Number of participants Mean age (years)	2569 60.8	3991 63.8	2647 61	2289 63.3	2737 68.7
Duration of follow-up	Mean 41.4 months	Mean 1 year	1.3 yrs	Mean 10.4 months	Median 21 months
Drug studied	Enalapril vs placebo	Metoprolol CR/XL vs placebo	Bisoprolol vs placebo	Carvedilol vs placebo	Eplerenone vs placebo
Inclusion criteria/ EF criterion for entry	Congestive HF with EF ≤ 35%	HF NYHA class II-IV and EF ≤ 40%	HF NYHA class III-IV and EF ≤ 35%	Severe chronic HF with EF < 25%	HF NYHA class II and EF ≤ 35%
Background HF treatment	Diuretics: 85% β-blockers: 8% Digitalis: 67% Vasodilators: 67%	Diuretics: 91% ACEi/ARBs: 96% Digitoxin: 64%	Diuretics: 99% ACE i: 96% Dihydropyridine Ca antagonists: 2% Nitrates: 58% Digoxin: 52%	Diuretics: 99% ACE i/ARBs: 97% Spironoloactone: 20% Digitalis: 66%	Diuretics: 84% β -blockers: 87% ACEi/ARBs or both: 93% Digitalis: 27%
Participants with diabetes n (%)	26%	984 (25%)	12%	26%	859 (31%)
Results in the overall population					
Primary endpoint	All-cause death: RR 16% (95% CI 5.26), *p* < 0.0036	All-cause death: HR 0.66 (0.53, 0.81), *p* = 0.00009	All-cause death: HR 0.66 (0.54, 0.81), *p* < 0.0001	All-cause death: RR 35% (18, 48%), *p* = 0.00013	CV death or hospitalization for HF: HR 0.63 (0.54, 0.74), *p* < 0.001
Secondary endpoints	All-cause death or hospitalization for HF: RR 26% (95% CI 18, 34), *p* < 0.0001	CV death: HR 0.62 (0.50, 0.78), *p* = 0.0003 All-cause death or hospitalization for HF: RR 31% (20%, 40%), *p* < 0.001 Hospitalization for HF: 10% (met) vs 14.7% (pbo), *p* < 0.001	CV death: HR 0.71 (0.56–0.90) *p* = 0.0049 Exploratory endpoint: hospitalization for HF. HR 0.64 (0.53–0.79) *p* < 0.0001	Death or hospitalization for any reason: RR 24% (13, 33%), *p* < 0.001	CV death: HR 0.76 (0.47, 0.70), *p* = 0.01 Hospitalization for HF: HR 0.58 (0.47, 0.70), *p* < 0.001
Results in the diabetes subpopulation					
Primary endpoint	All-cause death: Non-significant *p* for interaction	All-cause death: HR 0.82 (0.56, 1.19), *p* > 0.2	All-cause death: HR 0.81 (0.51, 1.28)	All-cause death: RR 35% (16, 50%)	CV death or hospitalization for HF: HR 0.541 (0.418, 0.699), *p* < 0.0001. p for interaction: 0.10
Secondary endpoint	All-cause death or hospitalization for HF Non-significant p for interaction	HF hospitalization: RR 37% (53%, 15%; p = 0 .0026	NR	NR	NR
References	[13, 17]	[20, 21]	[71, 72]	[73, 74]	[25, 26]

*HF* heart failure, *EF* ejection fraction, *DM* diabetes mellitus, *RR* relative risk, *HR* hazard ratio, *CV* cardiovascular, *HR* hazard ratio,

*Morbidity defined as defined as incidence of cardiac arrest with resuscitation, hospitalization for HF, receipt of i.v. inotropic or vasodilatator therapy for ≥ 4 h

**Table 2:** Key features, DM prevalence, and treatment effect on HF outcomes in the overall study population and by prevalent DM in the large clinical HF trials involving digoxin, ARBs, ivabradine, and ARNI

Study	DIG trial	Val-HeFt	CHARM overall study program	SHIFT	PARADIGM-HF
Year completed	1997	2001	2003	2010	2014
Number of participants Mean age (yrs)	6800 63.5	5010 62.7	7599 66.0	6505 60.4	8399 63.8
Duration of follow-up	Mean 37 months	Mean 23 months	Median 37.7 months	Median 22.9 months	Median 27 months
Drug studied	Digoxin vs placebo	Valsartan vs placebo	Candesartan vs placebo	Ivabradine vs placebo	LCZ696 vs enalapril
Inclusion criteria/ EF criterion for entry	HF with EF ≤ 45% and sinus rhythm	HF NYHA class II-IV and EF < 40%	EF > 40%➔Preserved EF ≤ 40% with ACE inhibitor➔Added EF ≤ 40% with ACE inhibitor intolerance➔ Alternative	Chronic HF with EF ≤ 35% and sinus rhythm with heart rate ≥ 70beats per minute	HF NYHA class II-IV and EF ≤ 40%
Background HF treatment	Diuretics: 82% ACEi: 94% Nitrates: 43% Other vasodilators: 1%	Diuretics: 85% β -blockers: 35% ACEi: 93% Spironolactone: 5% Digoxin: 67%	Diuretics: 83% β-blockers: 55% ACEi: 41% Spironolactone: 17% Ca antagonists: 20% Digoxin/digitalis glycosides: 43% Other vasodilators: 38%	Diuretics: 83% β-blockers:89% ACEi: 78% ARBs:14% Aldosterone antagonists: 60% Cardiac glycosides: 22%	Diuretics: 80% β-blockers: 93% Mineralocorticoid antagonists: 55% Digitalis: 30%
Participants with diabetes n (%)	1933 (28%)	25%	2160 (28%)	1979 (30%)	2907 (35%)
Results in the overall population					
Primary endpoint	All-cause death: HR 0.99 (0.91, 1.07), *p* = 0.80	All-cause death: HR 1.02 (0.88, 1.18), *p* = 0.80 Mortality and morbidity*: HR 0.87 (0.77, 0.97), *p* = 0.009	All-cause death: HR 0.91 (0.83, 1.00), *p* = 0.055	CV death or hospitalization for HF: HR 0.82 (0.75, 0.90), *p* < 0.0001	CV death or hospitalization for HF: HR 0.80 (0.73, 0.87), *p* < 0.001
Secondary endpoints	CV death: HR 1.01 (0.93, 1.10), *p* = 0.78 Hospitalization for HF: HR 0.72 (0.66, 0.79), *p* < 0.001 CV death or hospitalization for HF: HR 0.85 (0.79, 0.91), *p* < 0.001	Hospitalization for HF: RR 27.5%, *p* < 0.001	CV death or unplanned admission for HF:HR 0.84 (0.77, 0.91), *p* < 0.0001 CV death: HR 0.88 (0.79, 0.97), *p* = 0.012	All-cause death: HR 0.90 (0.80, 1.02), *p* = 0.092 Hospitalization for HF: HR 0.74 (0.66, 0.83), *p* < 0.0001	CV death: HR 0.80 (0.71, 0.89), *p* < 0.001 Hospitalization for HF: HR 0.79 (0.71, 0.89), *p* < 0.001
Results in the diabetes subpopulation					
Primary endpoint	All-cause death: HR 1.04 (0.91, 1.20), *p* = 0.40	Mortality and morbidity*: non-significant interaction	NR	CV death or hospitalization for HF: HR 0.81 (0.69, 0.95), *p* for interaction 0.861	CV death or hospitalization for HF: HR 0.87 (0.77, 0.98), *p* for interaction: 0.40
Secondary endpoint	CV death: HR 1.06 (0.92, 1.24), *p* for interaction: 0.47 Hospitalization for HF: HR 0.79 (0.68, 0.91), p for interaction: 0.14 CV death or hospitalization for HF: HR 0.90 (0.80, 1.01), *p* for interaction: 0.27	NR	CV death or unplanned admission for HF: *p* for interaction 0.09	Hospitalization for HF: 0.71 (0.59, 0.86), *p* = 0.001	Hospitalization for HF: HR 0.79 (0.67, 0.94)
References	[18, 19]	[75]	[22]	[24]	[27]

*HF* heart failure, *EF* ejection fraction, *DM* diabetes mellitus, *RR* relative risk, *HR* hazard ratio, *CV* cardiovascular, *HR* hazard ratio,

*Morbidity defined as defined as incidence of cardiac arrest with resuscitation, hospitalization for HF, receipt of i.v. inotropic or vasodilatator therapy for ≥ 4 h

### Interventions addressing HF outcomes in patients with T2DM

#### Non-glycemic interventions

**Fig. 2:**
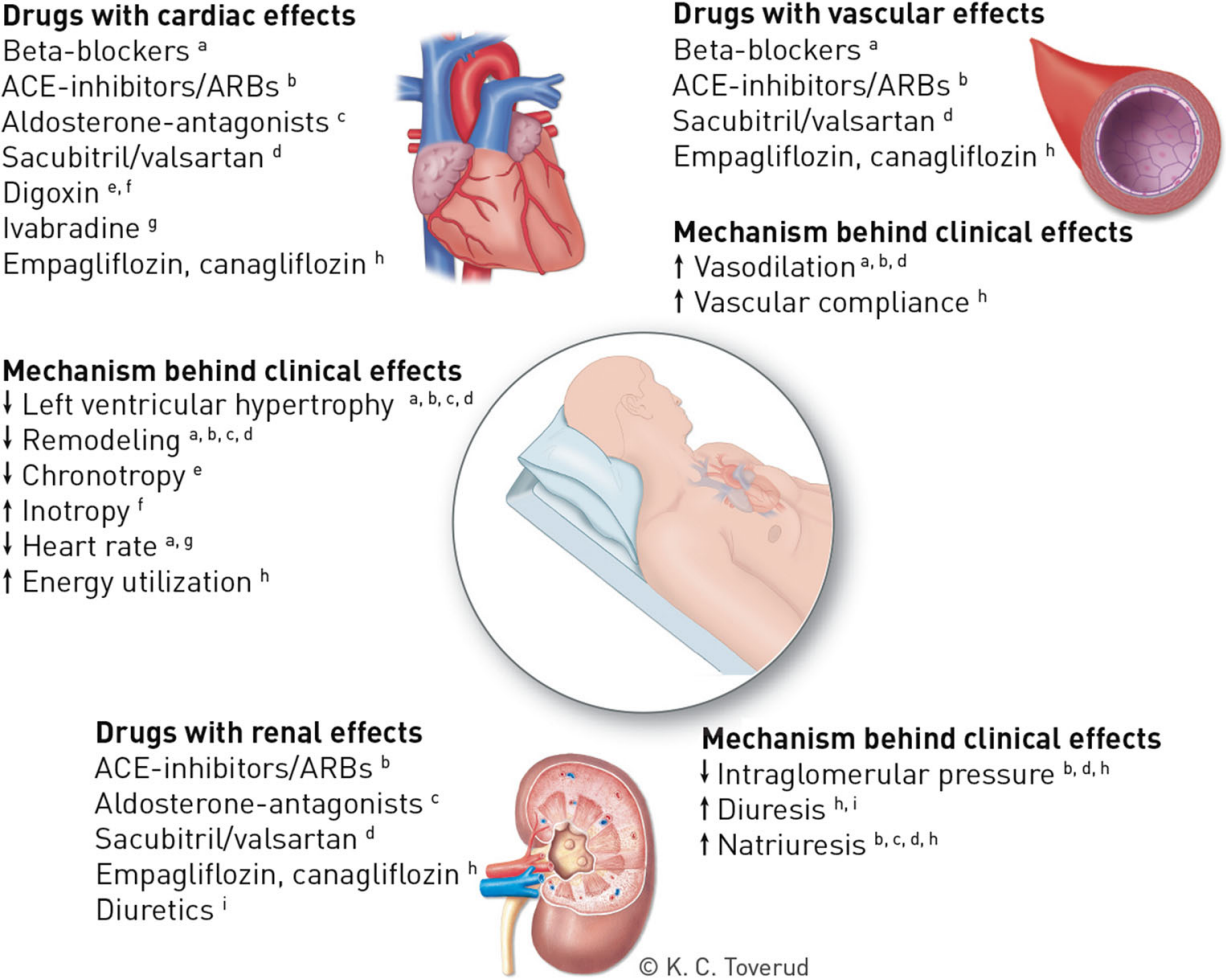
Overview over the mechanisms behind the clinical effects of evidence-based pharmacological treatment for prevention or treatment of HF, or HF-related events, in T2DM. Printed with permission from *©* Kari C. Toverud. Abbreviations: HF: heart failure, T2DM: type 2 diabetes, ACE: angiotensin converting enzyme, ARB: angiotensin receptor blocker

**Fig. 3:**
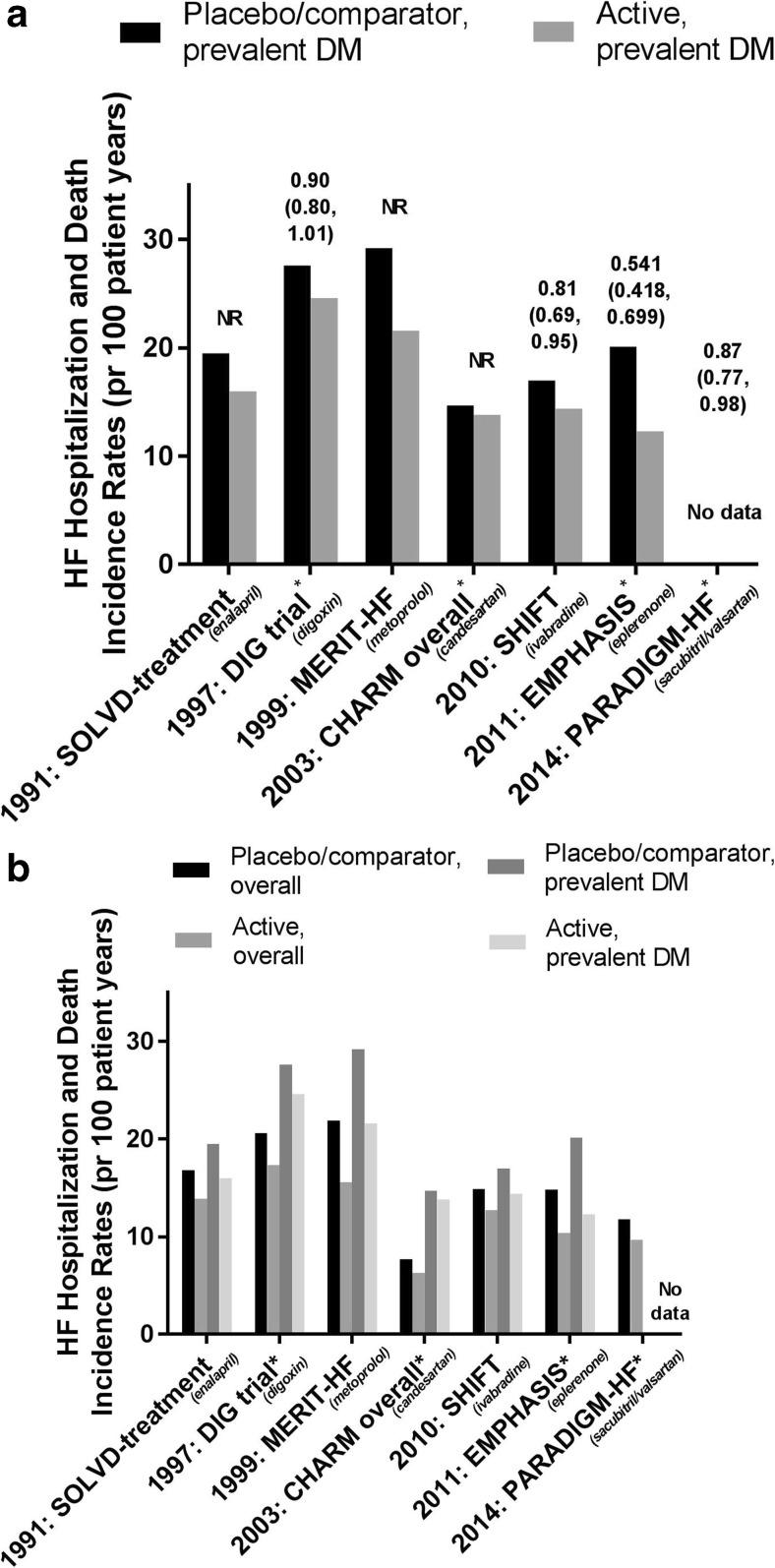
**a** Incidence rates of HF hospitalization and death in patients with T2DM participating in HF trials of different HF interventions (ACEi [13, 17], digoxin [18, 19], β-blocker [20, 21], ARB [10, 22], I_f_-blocker [23, 24], MRA [25, 26], and ARNI [27, 28]) and their hazard ratios (95% confidence interval). *: composite outcome comprises HF hospitalization and CV death. Abbreviations: HR: hazard ratio, HF: heart failure, NR: not reported, SOLVD: Studies of Left Ventricular Dysfuction, DIG-trial: The Digitalis Investigation Group (DIG) trial, MERIT-HF: Metoprolol CR/XL Randomized Intervention Trial in Congestive Heart Failure, CHARM: Candesartan Assessment of Reduction in Mortality and morbidity, SHIFT: The Systolic Heart Failure Treatment With the *I*_f_ Inhibitor Ivabradine Trial, EMPHASIS: Eplerenone in Mild Patients Hospitalization and Survival Study in Heart Failure, PARADIGM-HF: Prospective Comparison of ARNI with ACEI to Determine Impact on Global Mortality and Morbidity in Heart Failure. **b** Incidence rates of HF hospitalization and death in patients participating in HF trials of different HF interventions (ACEi [13, 17], digoxin [18, 19], β-blocker [20, 21], ARB [10, 22], I_f_-blocker [23, 24], MRA [25, 26], and ARNI [27, 28]) in the overall study population and in the subgroup with prevalent DM at baseline. *: composite outcome comprises HF hospitalization and CV death. Abbreviations: HR: hazard ratio, HF: heart failure, NR: not reported, SOLVD: Studies of Left Ventricular Dysfuction, DIG-trial: The Digitalis Investigation Group (DIG) trial, MERIT-HF: Metoprolol CR/XL Randomized Intervention Trial in Congestive Heart Failure, CHARM: Candesartan Assessment of Reduction in Mortality and morbidity, SHIFT: The Systolic Heart Failure Treatment With the *I*_f_ Inhibitor Ivabradine Trial, EMPHASIS: Eplerenone in Mild Patients Hospitalization and Survival Study in Heart Failure, PARADIGM-HF: Prospective Comparison of ARNI with ACEI to Determine Impact on Global Mortality and Morbidity in Heart Failure

The recommended treatment for HF in DM (symptomatic or to prevent HF hospitalization and/or death) is similar to treatment of HF in general and includes ACEis, β-blockers, MRAs, ARBs, and diuretics. Ivabradine or ARNI should be considered in the case of persistent symptoms and EF < 35%, and digoxin may be considered in patients with sinus rhythm and persistent symptoms. The mechanisms for clinical effects of these interventions are shown in Fig. 2. There is so far no evidence for a different treatment response in patients with or without DM in the large HF trials (*n* = 38,600 patients, Tables 1 and 2 and Fig. 3a, b), which means that the absolute benefit in patients with DM is greater due to increased risk of HF events. Side effects are also similar, except for increased risk of hyperkalemia with some agents blocking the RAAS system [3, 25, 76]. However, these data are, in lack of dedicated HF trials in T2DM patients, derived from subgroup analyses which have intrinsic limitations in terms of generalizability. Interpretation may therefore be challenging, in particular since the T2DM population often differs compared to the non-T2DM population in disease duration, metabolic control and vascular disease burden. Some HF studies furthermore report heterogeneity for the effect on HF hospitalizations and mortality with respect to geographical region [8, 77], likely explained by a multitude of factors including differences in the approach to diagnosis and etiology, availability of resources, and social and cultural circumstances [78].

#### HFpEF—some considerations

Of note, to date, no treatments have proven to be effective in reducing morbidity and mortality in HFpEF, and guidelines recommend symptomatic treatment of fluid overload with diuretics in addition to controlling risk factors such as blood pressure and atrial fibrillation [3]. Since there also are fewer studies conducted in HFpEF as compared to HFrEF, recommendations of HF treatments are mainly based on evidence from studies in patients with HFrEF (Tables 1 and 2). The “CHARM-preserved” and “I-preserved” studies found no benefit on mortality from the ARBs candesartan and irbesartan in HFpEF and only a moderate benefit on hospitalization for HF [79, 80]. Approximately 27% of the study population in both trials had T2DM with no heterogeneity in the results [22]. The Treatment of Preserved Cardiac Function Heart Failure with an Aldosterone Antagonist (TOPCAT) trial explored the effect of spironolactone on a primary outcome of CV death, aborted cardiac arrest and hospitalization for HF in a population with EF ≥ 45% of which approximately 30% had DM, with no significant effects on the primary outcome [81]. One discussed explanation for the neutral effect was the regional differences observed with higher event rates and significant effect in patients included in the Americas and lower event rates and no treatment effect in those included in Russia/Georgia, potentially caused by different practice patterns and use of hospitalization [8]. Three other smaller pilot studies with digoxin, perindopril, and carvedilol in HFpEF also failed to show any beneficial impact on survival or HF hospitalizations [82–84]. Sacubitril/valsartan (ARNI) is a new treatment option which combines an ARB with a neprilysin inhibitor and now is being studied in patients with symptomatic HFpEF (EF ≥ 45%) in the “Prospective comparison of ARNI with ARB Global Outcomes in heart failure with preserved ejection fraction” (PARAGON-HF) [https://clinicaltrials.gov/ct2/show/NCT01920711?term=PARAGON&rank=4.] which will be informative for its use also in this patient group.

#### Renin-angiotensin-aldosterone system inhibition

A dysregulated RAAS is a characteristic of both HF and T2DM, and RAAS inhibition is a recommended therapy in both conditions. Studies indicate that ACEis have a similar magnitude of effect on reducing mortality and HF hospitalization in populations with HF and prevalent T2DM as in those without T2DM [17, 85]. A post hoc analysis of the Studies of Left Ventricular Dysfunction (SOLVD) treatment and prevention studies illustrated this nicely showing that there were no interactions by the number and type of non-cardiac comorbidities (including T2DM) regarding treatment effects of enalapril on outcomes [86] (Table 1 and Fig. 3a, b). Enalapril furthermore slowed LV remodeling and the development of, and hospitalization for, HF when given to asymptomatic patients with LV dysfunction (EF ≤ 35%) [87].

MRAs reduce symptoms and mortality in both mild and severe symptomatic HF, with consistent effects also in the DM subgroups [26, 88] (Table 1, Fig. 3a, b), and are recommended as add-on therapy to β-blocker and an ACEi/ARB if persisting HF symptoms.

ARBs are less investigated, but have proven similar effects on mortality and HF hospitalization as ACEis when given as an alternative in ACEi-intolerant patients [89] (Table 2, Fig. 3a, b). They are therefore recommended as an alternative to, but not as an add-on to, ACEis, since they have not shown consistently to provide synergistic effects in reducing mortality [22, 75, 90].

Due to compensatory renin activation under treatment with ACEis or ARBs, it was postulated that a dual RAAS blockade with a renin inhibitor in combination, would improve cardiorenal outcomes in a T2DM population. In the Aliskiren Trial in Type 2 Diabetes Using Cardiorenal Endpoints (ALTITUDE), a study of patients with T2DM and kidney disease of whom 11% had a history of chronic HF, aliskiren increased the risk of side effects (in particular hyperkalemia) without benefit on outcomes, and hence renin inhibition is not recommended for patients with T2DM on ACEi or ARBs [76].

#### Beta (β) blockers

β-Blockers are, together with ACEis and MRAs, the cornerstones in the treatment of HF. Several large randomized clinical studies have reported reduced rates of mortality and hospitalization for HF with β-blockers in patients with HFrEF [20, 72, 73, 91], with no significant heterogeneity according to diabetes status (Table 1, Fig. 3a, b). A meta-analysis confirmed these findings in T2DM subjects [92]. Although carvedilol in one study was suggested to improve survival as compared to metoprolol, both in the overall, as well as in the patients with prevalent T2DM [93, 94], guidelines do not suggest a preferred β-blocker [1, 3]. β-Blockers are however still, potentially, underutilized in T2DM, perhaps due to fear of side effects, in particular blunting of symptoms of hypoglycemia. Post hoc analyses of the large β-blocker trials have furthermore reported some regional differences with smaller survival benefit in patients included in North America than in the rest of the world [77, 95]; however, these findings remain to be confirmed in prospective studies powered to explore geographical variations in treatment response.

#### Diuretics

Diuretics relieve symptoms of fluid retention in HF [96], but the effect on mortality is not established. Diuretics, loop or thiazide, are indicated when HF is decompensated with symptoms of fluid retention.

#### Digoxin

Digoxin/cardiac glycosides have been widely used in HF patients, both in the setting of sinus rhythm and atrial fibrillation, with or without prevalent DM. The Digitalis Investigation Group (DIG) trial included patients with HF with EF ≤ 45% and sinus rhythm, of which 28% had prevalent DM (Table 2) [97]. The overall study results showed no impact on mortality, but a reduction in the hospitalization for HF [18], which was confirmed in a later meta-analysis [98]. Subgroup analyses from the DIG trial showed that the effect tended to be more pronounced in the subgroups with the most severe HF (EF < 25%, NYHA class III or IV) [18, 99]. The subgroup by DM analyses were recently published and showed no significant interactions for any of the outcomes [19]. Of note is that very few patients used β-blockers and no patients used MRAs in the DIG trial; thus, the effect on outcomes from digoxin used on top of current standard of care is not known.

#### Angiotensin receptor-neprilysin inhibitor

Sacubitril inhibits neprilysin endopeptidase, blocking the catabolism of natriuretic peptides and other vasoactive peptides and thereby increasing their bioavailability (Fig. 2). The combination of valsartan and sacubitril was studied in the Prospective Comparison of ARNI with ACEI to Determine Impact on Global Mortality and Morbidity in Heart Failure (PARADIGM-HF) Trial involving HFrEF patients only [27]. The trial reported a reduced risk for CV death and hospitalization for HF for ARNI when compared to treatment with enalapril in symptomatic patients with HFrEF (Table 2) [27]. The results were consistent across subgroups, including the subgroup with T2DM comprising approximately 35% of the study population (Table 2 and Fig. 3a, b). Sacubitril/valsartan is now recommended in the HF guidelines of the ESC as a replacement for an ACEi to further reduce the risk of HF hospitalization and death in ambulatory patients with HFrEF who remain symptomatic despite optimal treatment with an ACEi, a β-blocker, and an MRA [3].

#### Ivabradine

Ivabradine, an inhibitor of the cardiac pacemaker current I_f_, reduces heart rate thereby reducing the cardiac work burden (Fig. 2). The Systolic Heart Failure Treatment With the *I*_f_ Inhibitor Ivabradine Trial (SHIFT) included HFrEF patients with sinus rhythm and a heart rate of at least 70 beats per minute (bpm) and demonstrated significant improvements in both the composite endpoint of HF hospitalization and CV death, and for HF hospitalization alone, with a similar magnitude of effect in those with prevalent DM [23] (Table 2 and Fig. 3a, b). Ivabradine is recommended if HF symptoms persist despite treatment with a β-blocker, ACEi, and MRA (in patients with sinus rhythm > 70 bpm).

### Glycemic interventions

**Fig. 4:**
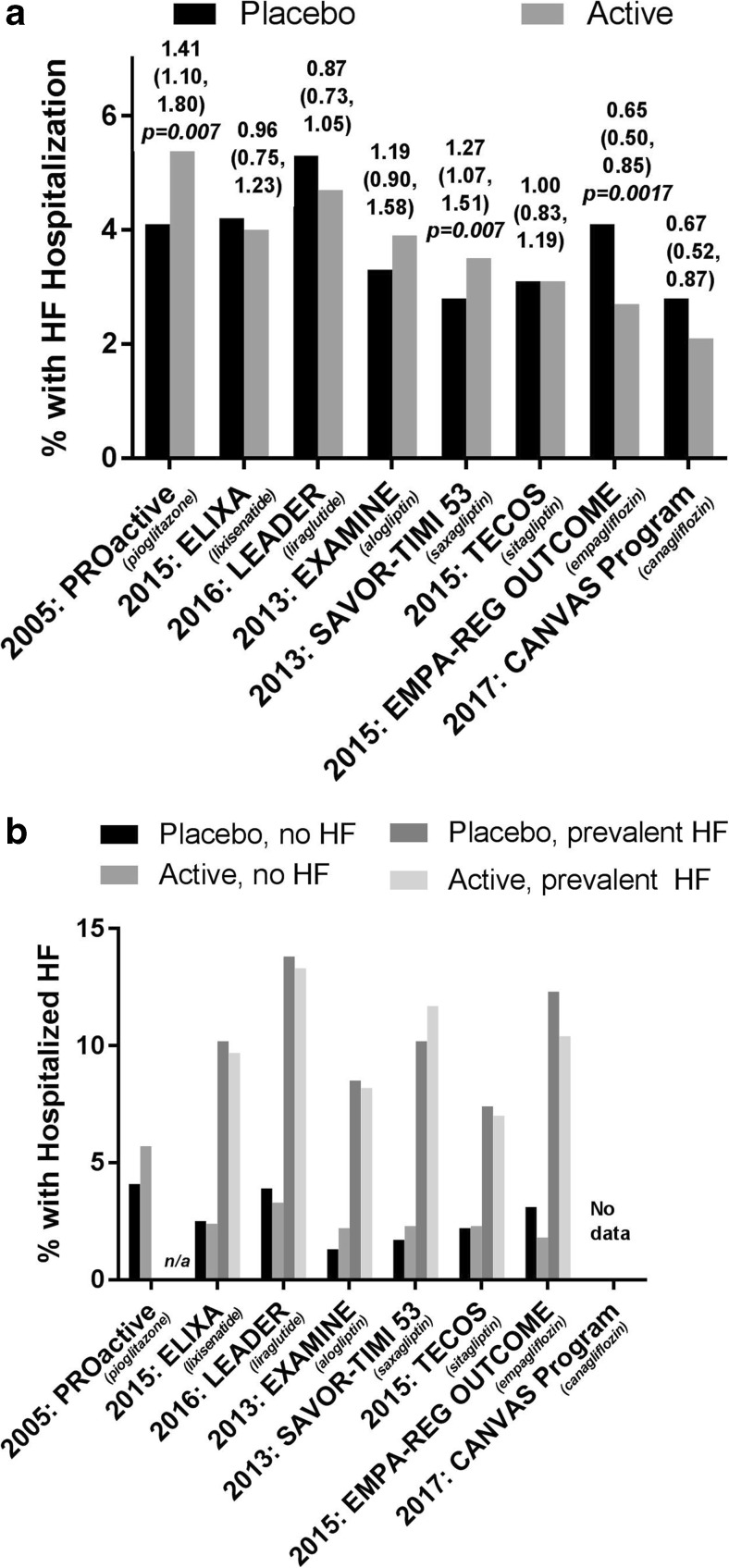
**a** Proportion of patients with HF hospitalization in the active and placebo arm in large CV outcome trials of different glucose-lowering drugs and their hazard ratios (95% confidence interval). Abbreviations: HF: heart failure, CV: cardiovascular, PROactive: PROspective pioglitAzone Clinical Trial In macroVascular Events, EXAMINE: The Examination of Cardiovascular Outcomes with Alogliptin versus Standard of Care, SAVOR-TIMI 53: The Saxagliptin Assessment of Vascular Outcomes Recorded in Patients with Diabetes Mellitus 53 trial, TECOS: The Trial Evaluating Cardiovascular Outcomes With Sitagliptin, ELIXA: the Evaluation of LIXisenatide in Acute Coronary Syndrome trial, LEADER: the Liraglutide Effect and Action in Diabetes: Evaluation of cardiovascular outcome Results—a long term evaluation trial, CANVAS: CANagliflozin cardioVascular Assessment Study. **b** Proportion of patients with HF hospitalization in the active and placebo arm according to presence of HF at baseline in large CV outcome trials of different glucose-lowering drugs. Abbreviations: HF: heart failure, CV: cardiovascular, n/a: not applicable, PROactive: PROspective pioglitAzone Clinical Trial In macroVascular Events, EXAMINE: The Examination of Cardiovascular Outcomes with Alogliptin versus Standard of Care, SAVOR-TIMI 53: The Saxagliptin Assessment of Vascular Outcomes Recorded in Patients with Diabetes Mellitus 53 trial, TECOS: The Trial Evaluating Cardiovascular Outcomes With Sitagliptin, ELIXA: the Evaluation of LIXisenatide in Acute Coronary Syndrome trial, LEADER: the Liraglutide Effect and Action in Diabetes: Evaluation of cardiovascular outcome Results—a long term evaluation trial, CANVAS: CANagliflozin cardioVascular Assessment Study

Despite a dose-dependent epidemiological association between glycemia and the risk of HF [45, 46], studies have failed to show reduced risk of HF and HF-related outcomes with strict glucose lowering, as confirmed in meta-analyses [100, 101]. Of interest is also that improving and maintaining glycemic control does not seem to contribute to prevent the progression of cardiac dysfunction in T2DM [102]. Glucose-lowering treatment modalities are however of importance, since different drugs have different impact on the risk of HF. In fact, evidence suggests an increased risk of hospitalization for, or precipitation of, HF with some classes of glucose-lowering drugs [37, 101, 103, 104] (Table 3) as will be discussed below. Of interest, two newer blood glucose-lowering drugs of the SGLT-2 inhibitor class, empagliflozin and canagliflozin, recently showed to reduce the risk of hospitalization for HF by 35% and 33%, respectively [41, 42, 105] (Fig. 4a, b), an effect likely related to improved hemodynamics induced by diuresis, transient natriuresis, and increased vascular compliance, with a subsequent reduction of loading of the myocardium (Fig. 2). Unlike some trials involving non-glycemic interventions for HF, till date CV outcomes trials of glycemic interventions in T2DM have not reported any geographical or racial heterogeneity in the effect on survival or HF hospitalizations [35, 38, 39, 42], although only few trials have been positive.

**Table 3:** Key features, HF prevalence, and treatment effect on first HF hospitalization by prevalent HF in contemporary CV outcomes trials of glucose-lowering drugs in T2DM

	PROactive		EXAMINE		SAVOR-TIMI 53		TECOS		ELIXA		LEADER		EMPA-REG OUTCOME		CANVAS	
Key incl. crit	Established CV disease		Acute coronary syndrome within 15–90 days		Established CV disease or high CV risk		Established CV disease or high CV risk		Acute coronary syndrome within 180 days		Established CV disease or high CV risk		Established CV disease		Established CV disease or high CV risk	
Key excl. crit	NYHA II-IV		NYHA IV		Se-Creat > 6 mg/dL		eGFR < 30 ml/min/1.73^2^		eGFR < 30 ml/min/1.73^2^		NYHA IV		eGFR < 30 ml/min/1.73^2^		eGFR≤ 30 ml/min/1.73^2^	
	Pio	Pbo	Alo	Pbo	Saxa	Pbo	Sita	Pbo	Lixi	Pbo	Lira	Pbo	Empa	Pbo	Ca	Pbo
Mean																
age (yrs)	61.9	61.6	61.0	61.0	65.1	65.0	65.4	65.5	59.9	60.6	64.2	64.4	63.1	63.2	na	63.4
T2D Duration* (yrs)	8	8	7.1	7.3	10.3	10.3	11.6	11.6	9.2	9.4	12.8	12.9	57.4%	57.0%	63.2	13.7
HbA1c, %	7.8	7.9	8.0	8.0	8.0	8.0	7.2	7.2	7.7	7.6	8.7	8.7	8.1	8.1	13.5	8.2
Trial duration^#^	34.5 months		18 months		2.1 yrs		3.0 yrs		25 months		3.8 yrs		3.1 yrs		126.1 weeks	
*n*	5238		5380		16,492		14,671		6068		9340		7020		10,142	
Rand.	1:1		1:1		1:1		1:1		1:1		1:1		2:1		2:1, 1:1¤	
Dosages studied	Pio 15.30 or 45 mg		Alo 6.25, 12.5 or 25 mg		Saxa 2.5 or 5 mg		Sita 50 or 100 mg		Lixi 10 or 20 μg		Lira 0.6, 1.2 or 1.8 mg		Empa 10 or 25 mg		Cana 100 or 300 mg	
Treatment effects on HF hospitalization in overall group																
	Pio	Pbo	Alo	Pbo	Saxa	Pbo	Sita	Pbo	Lixi	Pbo	Lira	Pbo	Empa	Pbo	Cana	Pbo
*N*	2605	2633	2701	2679	8210	8212	7332	7339	3034	3034	4668	4672	4687	2333	5795	4347
% (*n*) HFH	5.7% (149)	4.1% (108)	3.9% (106)	3.3% (89)	3.5% (289)	2.8% (228)	3.1% (228)	3.1% (229)	4.0% (122)	4.2% (127)	4.7% (218)	5.3% (248)	2.7% (126)	4.1% (95)	2.1% (123)	2.8% (120)
HR (95% CI) HFH	1.41 (1.10, 1.80), *p* = 0.007		1.19 (0.9, 1.58) *p* = 0.220		1.27 (1.07, 1.51) *p* = 0.007		1.00 (0.83, 1.19) *p* = 0.95		0.96 (0.75, 1.23) *p* = 0.75		0.87 (0.73, 1.05) *p* = 0.14		0.65 (0.50, 0.85) *p* = 0.0017		0.67 (0.52–0.87)	
Treatment effects on HF hospitalization in subgroup without prevalent HF																
% (*n)* without prevalent HF	100% (2605)	100% (2633)	71.5% (1930)	71.6% (1917)	87.2% (7154)	87.2% (7163)	82.2% (6029)	81.7% (5999)	77.5% (2352)	77.7% (2358)	86.0% (4015)	86.0% (4020)	90.1% (4225)	89.5% (2089)	86.1% (4992)	84.9% (3689)
% (n) HFH	5.7% (149)	4.1% (108)	2.2% (42)	1.3% (23)	2.3% (165)	1.7% (126)	2.2% (131)	2.3% (135)	2.4% (56)	2.5% (58)	3.3% (131)	3.9% (90)	1.8% (78)	3.1% (65)	Not reported	
HR (95% CI) HFH	1.41 (1.10, 1.80)		1.76 (1.07, 2.90)		1.32 (1.04, 1.66)		0.96 (0.76, 1.23)		0.89 (0.67, 1.40)		0.82 (0.65, 1.04)		0.59 (0.43, 0.82)		Not reported	
Treatment effects on HF hospitalization in subgroup with prevalent HF																
% (*n*) with prevalent HF	N/A	N/A	28.5% (771)	28.4% (762)	12.8% (1056)	12.8% (1049)	17.8% (1303)	18.3% (1340)	22.5% (682)	22.3% (676)	14.0% (653)	14.0% (652)	9.9% (462)	10.5% (244)	13.9% (803)	15.1% (658)
% (*n*) HFH	N/A	N//A	8.2% (63)	8.5% (65)	11.7% (124)	10.2% (102)	7.4% (97)	7.0% (94)	9.7% (66)	10.2% (69)	13.3% (87)	13.8% (90)	10.4% (48)	12.3% (30)	Not reported	
HR (95%CI) HFH	N/A		1.00 (0.71, 1.42) *p* = 0.996		1.21 (0.99, 1.58) *p* = 0.15		1.03 (0.77, 1.36)		0.93 (0.66, 1.30)		0.95 (0.71, 1.28)		0.75 (0,48, 1.19)		Not reported	
Reference	[29, 30]		[35, 36]		[37, 38]		[39, 40]		[31, 32]		[33, 34]		[41, 42]		[105]	
Abbreviations	*“median duration” given in PROactive and “% with > 10 years duration” given in EMPA-REG OUTCOME #: mean given in PROactive, all others are median ¤: CANVAS was 2:1 randomization, whereas CANVAS-R was 1:1 yrs – years, T2D – type 2 diabetes, cv – cardiovascular, HFH – heart failure hospitalization, Rand- randomization

#### Metformin

Metformin is the recommended first-line blood glucose-lowering treatment in T2DM patients with HF [3]. A previous contraindication for its use in HF, due to concerns for lactic acidosis, was removed by the FDA in 2007 [106] following retrospective studies reporting improved outcomes with lower re-admission and mortality rates as compared to other glucose-lowering treatments in patients with T2DM and HF [107–109]. A later, larger systematic review supported this conclusion [110]. No RCTs of metformin indicate, however, a role in the prevention of HF or HF outcomes.

#### Sulphonylureas

In patients with newly diagnosed T2DM enrolled in the UKPDS there was no increased risk of HF associated with the use of sulphonylurea [111], a finding also seen in the ADVANCE trial [112]. However, some retrospective studies have indicated that second generation sulphonylureas might be associated with 18–30% increased risk of HF as compared to metformin [113, 114]. One Canadian retrospective study, based on the Saskatchewan Health records, reported similar results (i.e., increased HF admission rates associated with sulphonylurea as compared to metformin), but when background characteristics (e.g., history of coronary heart disease, use of CV medication) were adjusted for, the difference was no longer significant [115]. The safety of sulphonylurea in HF populations with DM is thus not fully established and ongoing trials like the Italian Thiazolidinediones or Sulfonylureas and Cardiovascular Accidents Intervention Trial (TOSCA-IT) [116]), CARdiovascular Outcome Trial of LINAgliptin Versus Glimepiride in Type 2 Diabetes (CAROLINA®) trial [117, 118] and The Glycemia Reduction Approaches in Diabetes: A Comparative Effectiveness Study (GRADE) [119] might provide further insights in these matters.

#### Insulin

With the expanding armamentarium of non-insulin therapies for T2DM, insulin initiation typically occurs late in the T2DM disease trajectory [120]. Consequently, we observe a general pattern of a more deleterious cardiometabolic risk profile among patients initiating insulin [121] with a corresponding relative higher occurrence of CV events as compared to non-insulin users [122]. Mechanistically, since insulin therapy can induce weight gain [123], sodium retention, and fluid retention [124] and is being discussed to have some other vascular detrimental effects [125], it has been postulated that such therapy in T2DM potentially could worsen outcomes, particularly in a HF setting. However, in well powered RCTs assessing the effect on CV outcomes, including HF outcomes, from insulin-based intensive versus conventional glucose lowering, neither beneficial nor harmful effects on HF were observed, i.e., in the Outcome Reduction with an Initial Glargine Intervention (ORIGIN) trial where HF hospitalization HR was 0.91 (95% CI 0.87–1.05]) [126] and UKPDS where HF RR was 0.78 (95% CI 0.39, 1.55) [111]). Recently, a consistent result in the long-term follow-up of ORIGIN was also reported (HR 1.03 (95% CI 0.97, 1.10]) [127]. There are however no trials designed to test effects on CV outcomes, including HF, of insulin-treatment with the intent of achieving glycemic equipoise between treatment arms.

#### Thiazolidinediones

Thiazolidinediones (TZDs) are insulin sensitizing drugs known to cause fluid retention. The class includes rosiglitazone and pioglitazone, which both in dedicated outcome trials were associated with increased risk of HF hospitalizations, even though patients with a previous history of HF (NYHA class II-IV) were excluded; the Rosiglitazone evaluated for cardiovascular outcomes in oral agent combination therapy for type 2 diabetes (RECORD) study reported a HR of 2.10 (95% CI 1.35, 3.27) [128], and the PROspective pioglitAzone Clinical Trial In macroVascular Events (PROactive) a HR of 1.41 (95% CI 1.10, 1.80) [29] (Table 3, fig. 4a and b). Although the drugs might modulate the risk of MI differently [129], a meta-analysis indicated that they increase the risk of HF to a similar extent [130]. One study aiming to understand the cardiac dynamics with TZD therapy found that rosiglitazone significantly increased left ventricular end-diastolic volume [131]. The use of TZDs is contraindicated in patients with known or prior HF with functional class NYHA I-IV and if used, patients with known HF risk factors should be monitored for HF symptoms such as oedema and weight gain.

#### Dipeptidylpeptidase-4 (DPP4) inhibitors

The drug class of DPP4 inhibitors has accumulated solid evidence from RCTs on risk of HF, with three large CV outcome trials (Table 3) recently completed and reported, all with hospitalization for HF as a pre-specified secondary or exploratory outcome [36, 37, 40] (Table 3). The Saxagliptin Assessment of Vascular Outcomes Recorded in Patients with Diabetes Mellitus (SAVOR-TIMI) 53 trial compared saxagliptin and placebo on top of standard care in T2DM patients either with established CVD or at risk for CVD and found no difference in the primary outcome (a composite of CV death, MI, or ischemic stroke). However, patients in the saxagliptin group were at higher risk of being admitted to the hospital for HF (HR 1.27, 95% CI 1.07–1.51, *p* = 0.007) than were those in the placebo group (Fig. 4a). Interestingly, but not surprisingly, the increased risk was associated with increased levels of NT-proBNP and an estimated GFR ≤ 60 ml/min/1.73 m^2^ at baseline, regardless of treatment allocation [37], and in absolute term, patients with prevalent HF at baseline had a higher risk (Fig. 4b). However, the incremental HF signal with saxagliptin was most prominent in those without prevalent HF (HR 1.32 (95% CI 1.04, 1.66) vs HR 1.21 (95% CI 0.99, 1.58)) (Table 3 and Fig. 4b). The Examination of Cardiovascular Outcomes with Alogliptin versus Standard of Care (EXAMINE) study compared alogliptin to placebo in T2DM patients with a recent acute coronary syndrome [36], of which 28% had reported HF at inclusion. There was no difference between the groups in the risk of the primary outcome (composite of CV death, non-fatal MI, or non-fatal stroke), but there was a numerically increased risk of hospitalization for HF associated with alogliptin (HR 1.19 (95% CI 0.89, 1.58)) (Table 3). The risk of the primary outcome was consistent in the subpopulation with established HF at inclusion; however, as observed in SAVOR-TIMI53, a relative higher risk for HF hospitalization with alogliptin therapy was seen in patients without prevalent HF (HR 1.76 (95% CI 1.07, 2.90)) as compared to those with HF (HR 1.00 (95% CI 0.71, 1.42)) (Fig. 4b). The Trial Evaluating Cardiovascular Outcomes With Sitagliptin (TECOS) investigated the effect of sitagliptin vs placebo in T2DM patients with established CVD, with neutral effect on the primary outcome (CV death, non-fatal MI, non-fatal stroke, or hospitalization for unstable angina) [40]. There was no difference in the risk for hospitalization for HF between the treatment groups (HR 1.00 (95% CI, 0.83 to 1.20)), regardless of HF status at baseline. A mechanistic study exploring the impact of vildagliptin versus placebo on systolic function as measured by EF with echocardiography in 254 T2DM patients with HF in NYHA class I-III found no differences in change in EF after 1 year (primary outcome), but a larger increase in both LV end-diastolic and end-systolic volumes was seen with vildagliptin [132], an effect of similar magnitude as observed with rosiglitazone therapy [131]. Finally, although no dedicated outcome study has been reported yet for linagliptin, a meta-analysis of pooled registration studies indicated no increased risk of hospitalization for HF [133].

Thus, the published trials so far with DPP4 inhibitors suggest that the increased risk of HF seen with certain class members does not represent a class effect. In the coming years two CV outcome trials with linagliptin will be reported, i.e., the CAROLINA® trial [117, 118] (linagliptin vs sulphonylurea) in 2019 and the CArdiovascular Safety & Renal Microvascular outcomE study with LINAgliptin (CARMELINA®) (linagliptin vs placebo) in 2018 [https://clinicaltrials.gov/ct2/show/NCT01897532?term=CARMELINA&rank=1.].

#### GLP-1 receptor agonists

GLP-1 receptor agonists are indicated to reduce glucose in T2DM and belong to the incretin class of drugs. Apart from glucose-lowering effects, they also have a number of non-glycemic effects including reducing appetite (and inducing weight loss), modestly reducing BP and increasing pulse rate. Two sufficiently powered CV outcomes trials within this drug class have thus far reported; the Evaluation of LIXisenatide in Acute Coronary Syndrome (ELIXA) trial and the Liraglutide Effect and Action in Diabetes: Evaluation of cardiovascular outcome Results—a long term evaluation (LEADER) trial [31, 33]. The ELIXA trial (Table 3) included 6068 T2DM patients with recent acute coronary syndrome (< 180 days) and reported a neutral effect on both the primary outcome (composite of CV death, non-fatal stroke, non-fatal MI and hospitalization for unstable angina) and on hospitalization for HF (HR 0.96 (95% CI, 0.75 to 1.23)) (Fig. 4a, b) [31]. In this large trial, only a slightly increased heart rate was observed with lixisenatide, with mean 0.4 bpm (95% CI 0.1, 0.6), a result potentially influenced by the high use of β-blockers in the study population (85 and 84% at baseline in the placebo and lixisenatide groups, respectively). The LEADER trial (Table 3) followed 9340 T2DM patients with established CVD (approximately 80%) or more than one CV risk factor (approximately 20%) for a median time of 3.8 years, and reported significantly decreased risk for the primary outcome (composite of CV death, non-fatal stroke and non-fatal MI) with liraglutide as compared to placebo (HR 0.87 (95% CI 0.78, 0.97)). This result was driven by a 22% relative reduction in CV death (HR 0.78 (95% CI 0.66, 0.93)), whereas non-fatal MI and non-fatal stroke were not significantly affected. Fourteen % of the study population had prevalent HF, and the drug had no impact on hospitalization for HF (HR 0.87 (95% CI 0.73, 1.05) Fig. 4a, b)) despite the slightly increased heart rate seen with liraglutide of 3 bpm relative to placebo. Lately, smaller studies have suggested potentially adverse effects on cardiac function of liraglutide: the Effects of Liraglutide on Clinical Stability Among Patients With Advanced Heart Failure and Reduced Ejection Fraction: A Randomized Clinical Trial (FIGHT) trial [134] randomized 300 adults (60% with T2DM) with acute decompensated HFrEF to 1.8 μg liraglutide or placebo. After 6 months, there was a numerically increased risk for death and hospitalization for HF (which were parts of a hierarchical primary endpoint together with NT-proBNP levels) with liraglutide, and this finding was accompanied by increased LV diastolic and systolic volumes. In this study, there was no difference in the change of heart rate, but there were more cases of arrhythmia with liraglutide reported as safety events (17 vs 11% in liraglutide and placebo). These findings were in line with the Effect of Liraglutide on Left Ventricular Function in Chronic Heart Failure Patients With and Without Type 2 Diabetes Mellitus (LIVE) study [135] including 241 patients with chronic HFrEF (approximately 30% had T2DM) where no impact on systolic function by echocardiography was seen, but a significantly increased heart rate with 6 bpm with liraglutide vs 1 bpm with placebo, *p* < 0.001. The mechanisms behind the increased heart rate and further effects on myocardial function with GLP-1 receptor analogues remain to be elucidated [136–138].

#### Sodium-glucose transporter (SGLT) 2 inhibitors

SGLT-2 inhibitors reduce glucose reuptake in the kidneys by inhibiting the SGLT-2 transport protein thereby causing glucosuria, urinary caloric loss, and volume loss (osmotic diuresis, transient natriuresis). Within this class of drugs, results from two sufficiently powered CV outcome trials have been reported, i.e., EMPA-REG OUTCOME® testing empagliflozin versus placebo and the CANVAS Program (CANagliflozin cardioVascular Assessment Study), testing canagliflozin versus placebo (Table 3). The EMPA-REG OUTCOME trial randomized and treated 7020 T2DM patients with established CV disease, of which 10% had prevalent HF, to assess CV safety of empagliflozin given on top of standard of care [41]. The primary outcome (CV death, non-fatal MI, and non-fatal stroke) was significantly reduced by 14%, driven by a reduction in CV death by 38%. In both the placebo and empagliflozin groups, the most frequent modes of CV death were sudden death, death from HF, and presumed CV death (death of unknown cause), and all categories of CV death contributed to the risk reduction with empagliflozin. Notably, hospitalization for HF was reduced by 35% (HR 0.65 (95% CI 0.50, 0.85)) with 4.1 and 2.7% of patients in placebo and empagliflozin groups being hospitalized with HF (Table 3, Fig. 4a, b), as was the composite of hospitalization for HF and CV death (HR 0.66 (0.55, 0.79)) and time to introduction of loop diuretics (0.62 (95% CI 0.53–0.73). Subgroup analyses revealed no significant heterogeneity with regards to baseline kidney function, CV medication used or prevalent HF [42]. Recent guidelines on the diagnosis and treatment of HF issued by the ESC recommend that the use of empagliflozin be considered (class IIa recommendation) in patients with T2DM to prevent or delay the onset of HF and prolong life [3], and FDA also recently approved empagliflozin to reduce the risk of CV death in adults with T2DM and established CV disease.

The CANVAS Program, combining data from two independent trials (the CANVAS trial and the CANVAS-R trial) [139], included patients with established CVD or being at high CV risk, of which approximately 14% had HF at baseline [105]. In this program, when compared to placebo, canagliflozin reduced the primary outcome (CV death, non-fatal MI, non-fatal stroke) by 14% (HR 0.86 (0.75–0.97) [105], but without any significant effect on CV or all-cause death. Hospitalization for HF was however reduced (HR 0.67 95% CI 0.52–0.87), with similar magnitude as with empagliflozin with 2.1 and 2.8% of the patients on canagliflozin and placebo, respectively, being hospitalized for HF during the program. At variance with the safety findings of empagliflozin, canagliflozin was associated with a significant increased risk for lower leg amputation and bone fractures [105].

There are several mechanisms potentially explaining the benefits of empagliflozin and canagliflozin on HF hospitalizations, one being the reduction in blood pressure, arterial stiffness, double product (also known as rate pressure product) and pre-load without any compensatory increase in heart rate [140, 141]. A reduction in weight and visceral fat may also play a role [142, 143], as may the reduction in uric acid and improved energy utilization, and for empagliflozin, an increase in hematocrit [41, 144]. Data from the EMPA-REG OUTCOME® trial and the CANVAS Program also revealed significantly decreased progression in nephropathy and risk of adverse renal outcomes (dialysis, doubling of s-creatinine, renal transplant) [145] which is likely due to the reduction in glomerular hypertension and restored tubule-glomerular feedback [146]. Thus, several mechanisms may contribute to the effects of empagliflozin and canagliflozin on HF outcomes [147–149]. CV outcomes trials with the other SGLT-2 inhibitors are due to be published within the next few years, all with hospitalization for HF as a pre-specified secondary outcome [https://clinicaltrials.gov/ct2/show/NCT01730534?term=declare+timi&rank=1., https://clinicaltrials.gov/ct2/show/NCT01986881?term=vertis&rank=1.].

In a meta-analysis of phase 2 and 3 trials with dapagliflozin there was no sign of increased risk of HF hospitalization as compared to placebo or comparator (HR 0.36 (95% CI 0.16, 0.84), but the analysis was based only on 26 events [150].

## Future research involving patients with DM and HF

There is a growing evidence base on how to manage and prevent HF in T2DM. The main lesson learned from contemporary clinical trials is that patients with concomitant T2DM and HF experience 1.9–4.3-fold higher rates of HF hospitalization than patients with either condition alone (Figs. 1a, b and 2). The incremental risk present in patients with co-existing HF and T2DM renders room for further improvement and refinement of treatment to prevent worsening of HF and death. One approach, which has been demonstrated and is recommended, aims at an intervention with a global risk factor approach (i.e., addressing hypertension, albuminuria, dyslipidemia, hyperglycemia, physical inactivity), which in a RCT proved to prevent deterioration in cardiac function in T2DM over 2 years [151]. Another approach is to implement the new evidence from recent RCTs (Figs. 3a, b and 4a, b).

Results from ongoing or planned dedicated HF studies (e.g., ongoing studies of ARNI [85], studies of empagliflozin in patients with chronic HFrEF and HFpEF with or without T2DM due to report in 2020) [https://clinicaltrials.gov/ct2/show/NCT03057951?term=EMPEROR&rank=2., https://clinicaltrials.gov/ct2/show/NCT03057977?term=EMPEROR&rank=1.], or studies of dapagliflozin [https://www.astrazeneca.com/media-centre/press-releases/2016/astrazeneca-announces-two-new-phase-IIIb-trials-for-Forxiga-in-chronic-kidney-disease-and-chronic-heart-failure-120920161.html.], or ongoing T2DM studies, will potentially shed further light over how best to manage this vulnerable group of patients.

## Summary

This review summarizes the literature on HF outcomes in patients with T2DM in studies involving guideline-recommended HF therapy studied in 38,600 patients as well as outcomes from contemporary trials of specific glucose-lowering drugs studied in 74,351 patients. The evidence base for HF management in the T2DM population stems mainly from subgroup analyses in HF trials and indicates similar magnitude of beneficial effect on symptoms and mortality as in the non-diabetic population. However, the absolute risk and event rates in patients with HF and T2DM are higher than in non-DM, signaling that there is room for significant improvements in the management of patients with T2DM and HF. The choice of blood glucose-lowering medication also seems to play a major role as some drugs (i.e., saxagliptin, TZDs) have a deleterious impact on the HF burden, whereas others, i.e., empagliflozin and canagliflozin, reduce the HF burden. Efforts to implement efficacious therapies are warranted, as well as further trials to better understand the pathophysiology of HF in T2DM.
